# The effects of topical (gel) astemizole and terfenadine on wound healing

**DOI:** 10.4103/0253-7613.43164

**Published:** 2008-08

**Authors:** D. Srikanth, Rekha R. Shenoy, C. Mallikarjuna Rao

**Affiliations:** Department of Pharmacology, Manipal College of Pharmaceutical Sciences, Manipal, Karnataka, India

**Keywords:** Astemizole, breaking-strength, epithelization, terfenadine, wound healing

## Abstract

**Objective:**

To develop topical gel preparations of astemizole and terfenadine and to investigate the actions of the gels on the healing of incision and excision wounds in male albino rats.

**Materials and Methods:**

Gels containing 1% astemizole, with varying concentrations of carbopol 934 (polymer), were prepared. Similarly, 1% terfenadine gels were made. The formulations were evaluated for release rate and stability. Incision and excision wounds were inflicted on male albino rats under ketamine anesthesia, taking aseptic precautions. The animals were divided into two groups. They were given a topical application of either astemizole or terfenadine gel, at a dose of 100 mg per wound, once daily, for 10 days in the case of incision wounds and till the time of complete closure in the case of excision wounds. On the 11^th^ day, breaking strength of the incision wound was measured. In the excision wound model, wound closure rate, epithelization time, scar features and hydroxyproline content of scar tissue were studied from the day of wounding till the day of the scab falling off, with no residual raw area.

**Results:**

Gels prepared using 0.8% carbopol 934 and 1% of drug in gel base were found to be stable. The gels of astemizole and terfenadine significantly (*P* < 0.05) promoted the phases of healing such as collagenation (in incision wounds), wound contraction and epithelization (in excision wounds).

**Conclusion:**

The gels of astemizole and terfenadine might play an important role in wound management program.

## Introduction

Wound healing is a complex and complicated process. It runs through a number of phases, which either run concurrently or are intimately interlinked through some chemical, biochemical and cellular pathways. A treatment could influence the healing of wound by intervening in any one or many phases of healing. No treatment, either systemic or local, could be considered inert on healing process,[1] unless it is proved experimentally.

Histamine is one of the mediators of acute inflammation. The exact contribution of histamine to repair the process of a wound is difficult to ascertain. However, histamine causes itching in the healing wound and the healed wound (scar). If itching is not contained in the healing wound, it may hamper the healing process or may cause damage to the scar. Doxepin, an antidepressant having anti-histaminic activity, upon topical use, has been shown to reduce the itching in burn wound and scars.[2] However, its influence on healing has not been studied. Thus, it is interesting to study the influence of topical antihistaminics on healing.

Astemizole and terfenadine were among the first nonsedating antihistaminic drugs to be introduced for therapeutic use. Subsequently their cardiac toxicity, e.g. ventricular fibrillation and torsades de pointes was discovered when they were given systemically, in high doses and in combination with CYP3A4 inhibitors. The occurrence of many such cases of cardiac toxicity led to a drastic reduction in their use. No attempt has so far been made to discover their topical utility, particularly in wound healing. Thus, the present work was aimed at studying a new use of these drugs in a new dosage form.

The study was, therefore, conducted on the healing of incision and excision wounds, using the gel preparations of terfenadine and astemizole.

## Materials and Methods

### Preparation of gels[3]

Formulation: Gels containing 1% of astemizole/terfenadine, with varying concentrations of carbopol (0.6%, 0.8% and 1.0%) were prepared. The gels were tested for stability, content uniformity and *in vitro* release through excised mouse skin. Gels containing 0.8% carbopol 934 and 1% of drug in gel base were finally selected for wound healing studies, as these were found to be more stable and had good release rates than the others. The compositions of these gels are as follows:

**Table:** 

Astemizole gel	Carbopol 934	80 mg
	Propylene glycol	1 ml
	Ethanol	2 ml
	Water	5.2 ml
	DMSO	1.5 ml
	Triethanolamine	*q.s.*
	Astemizole	100 mg
Terfenadine gel	Carbopol 934	80 mg
	Propylene glycol	1 ml
	Ethanol	2 ml
	Water	5.2 ml
	DMSO	1.5 ml
	Triethanolamine	*q.s.*
	Terfenadine	100 mg

### Stability studies

The gels were kept in collapsible tubes and were placed in the stability chamber, which was maintained at 40°C and 75% relative humidity for 90 days. The gels were then observed for any change in the consistency and color. Besides, the drug content uniformity was evaluated as described below.

### Content uniformity

Five hundred milligram of the gel was taken and dissolved in a small amount of buffer pH 7.4. The volume was made up with buffer. The absorbance was measured at 284 and 258 nm respectively, for astemizole and terfenadine.

### In vitro release studies through excised rat skin[4]

The rat skin soaked in buffer for 6-8 h was clamped carefully to one end of the dialysis cell (donor compartment). Fifty milliliter phosphate buffer saline (PBS) containing 20% ethanol was taken in a beaker (receptor compartment). Gel, of 1 g quantity, was spread uniformly on the membrane. The donor compartment was kept in contact with the receptor compartment. The temperature was maintained at 37 ± 0.1°C. At predetermined time intervals, 1 ml of the solution was pipetted out from the receptor compartment and immediately replaced with 1 ml of fresh PBS. The drug concentration of the acceptor fractions was determined against appropriate blank.

### Wound healing studies

Healthy inbred male albino rats of Wistar strain, weighing 180-200 g were selected. They were individually housed and maintained on normal food and water *ad libitum*, under standard living conditions of room temperature 25°C and day and night reverse cycle. The surgical interventions, under ketamine anesthesia (10 mg/kg), were carried out on the shaven back of the animals. Under each wound model of study, the wound bearing animals were divided into four groups, each group containing eight animals (n = 8). The first group of animals was left untreated and served as control. The second, third and fourth groups of animals received the topical applications, viz. gel base (base control), astemizole gel and terfenadine gel respectively, at approximately 100 mg per wound, once daily for 10 days in the case of incision wounds and a maximum of 20 days in the case of animals with excision wounds, starting from the day of surgery.

### Wound models

Incision wound: Two para-vertebral straight incisions of 6 cm each were made through the entire thickness of skin on either side, at least one cm lateral to the vertebral column, as per the methods described by Ehrlich and Hunt.[5] The sutures were removed on the 7^th^ day of wounding. The wound breaking strengths were measured on the 10^th^ day of wounding by continuous, constant water flow technique, as described by Lee.[6]

Excision wound: Excision wounds were made as described by Morton and Malone,[7] by excising the full thickness circular skin (approximately 500 mm^2^) from the nape of neck, under ketamine anesthesia. Wound contraction was assessed by tracing the wound area on polythene paper first and subsequently on a mm sq paper, every alternate day. The wound closure rate was expressed as percentage of the original wound size of 500 mm^2^ on selected days.

On day 15, an animal from each of the treated groups was sacrificed and the wound was excised and fixed in neutral buffered 10% formalin. Later, paraffin sections of 5 micron were made and stained with H & E stain for histopathological evaluation.

The number of days required for the scab to fall off without leaving a raw wound behind was considered the period of epithelization.

Healed scar on the day of epithelization was excised and used for determination of hydroxyproline content.[8]

The experimental protocols were approved by the Institutional Animal Ethics Committee of MAHE, Manipal.

### Statistical analysis

Results were analyzed using the one-way ANOVA, followed by *post hoc* test, viz. Scheffe's test using the Windows based SPSS computer package.

## Results

### Content uniformity

Both the gel formulations of terfenadine and astemizole showed 99% drug content uniformity in their formulations [Table 1].

**Table 1:** Content uniformity of astemizole and terfenadine gels

*Formulations*	*Percent drug ± S.D (n=3)*
Astemizole-gel	99.13±2.86
Terfenadine-gel	99.28±1.33

### Physical stability

Gels of terfenadine and astemizole, containing 0.8% carbopol 934 and 1% of drug were found to be stable in the accelerated stability studies, as there was no change in the color and consistency. Besides, air bubbles and crystals did not appear during the studies.

*In vitro* release studies of astemizole and terfenadine through rat skin

The release of the drugs from the formulations was progressive and steady. At the seventh hour, the rates of release of astemizole and terfenadine from their gel formulations were 71.30% and 67.85% respectively [Table 2].

**Table 2:** *In vitro* diffusion of astemizole and terfenadine from their gel formulations in Phosphate Buffer Saline (pH 7.4) containing 20% ethanol through rat skin

*Time in minutes*	*Cumulative percent drug release (Mean ± SD) (n=3) Astemizole-gel*	*Cumulative percent drug release (Mean ± SD) (n=3) Terfenadine-gel*
0	0	0
15	6.1±0.84	5.25±0.50
30	11.87±0.44	9.887±0.31
60	22.2±0.65	18.09±0.34
90	28.9±0.98	23.04±0.58
120	35.0±1.92	29.47±0.43
180	44.0±0.28	36.83±1.89
240	51.1±0.76	43.8±1.86
300	58.5±1.41	53.457±1.97
360	65.2±1.13	65.44±1.07
420	71.3±2.26	67.85±2.05

## Wound Healing Studies

### Incision wounds

The mean strength of the healed wound scar in control group of animals was 266 g. While the base of the gels did not influence the strength of the scar, both astemizole and terfenadine gels significantly increased (*P*< 0.05) the breaking strength as compared to control. The rate of increase was between 26% and 39 % respectively for astemizole and terfenadine [Table 3].

**Table 3:** Effect of topical application of astemizole and terfenadine gels in incision wound model

*Drug*	*Dose (mg/wound)*	*Breaking strength (g) Mean±S.E*	*% Increase in breaking strength compared to control*
Control	-	266.25±8.5	-
Gel base	100	271.64±2.61	1.8
Astemizole	100	334.12±7.9^a^^b^	26
Terfenadine	100	370.22±13.5^a^^b^	39
Allowance value*		38.19	

* Scheffe's test

a *P*<0.05 Vs control

b *P*<0.05 Vs gel base

n=8 in each group

### Excision wound

In control group, wound contracted to the extent of 3.7, 37, 74 and 91% by days 2, 6, 10 and 14. The gel base treatment, however, enhanced the wound closer rate on day 6 and 10. Wounds of the animals, which received astemizole, showed a similar rate of contraction as that of gel base on day 6 and day 10. However, these wounds contracted significantly faster than the gel base treated wounds on day 14. On the other hand, terfenadine gel showed significantly (*P*< 0.05) greater wound contraction, as compared to gel base on days 2, 6, 10 and 14 [Table 4].

**Table 4:** Effect of topical application of astemizole and terfenadine gels in excision wound model

*Groups*	*Dose (mg/wound)*	*Wound contraction (as % of original area 500 Sq mm) on day Mean ± S.E*				*Period of epithelization (Days) Mean ± S.E*	*Collagen (OHP) content of the scar (mg/g) Mean ± S.E*
				
		*2*	*6*	*10*	*14*		
Control	-	3.7±0.51	37.88±1.92	74.1±1.65	91.1±0.68	18.25±0.31	45.56±2.78
Gel base	100	6.1±1.69	48.96±1.62^a^	84.78±1.44^a^	93.23±0.99	17.87±0.31	45.77±1.8
Astemizole	100	5.83±0.94	54.38±2.71^a^	88.70±0.51^a^	97.95±0.23^a^^b^	16.37±0.18^a^^b^	58.95±1.72^a^^b^
Terfenadine	100	7.25±0.86	63.46±2.17^a^^b^	90.52±0.61^a^^b^	97.8±0.19^a^^b^	16.12±0.22^a^^b^	56.32±1.57^a^^b^
Allowance value*		8.19	8.97	4.88	2.45	1.10	8.66

* Scheffe's test

a *P*<0.05 Vs control

b *P*<0.05 Vs gel base

n=8 in each group

On day 15, in the control group, a small portion (less than 9%) of the original wound remained to be covered by the epidermis. Thus, these animals took a period of 18 days for complete epithelization. The gel base did not alter this. On the other hand, the gels of astemizole and terfenadine significantly (*P* < 0.05) promoted the epithelization process, thus shortening the period by two days [Table 4].

The collagen content in the healed scar of the control and the base applied wounds was around 45 mg/g of the tissue. The application of the gels of astemizole and terfenadine caused a significant (*P* < 0.05) increase in collagen content of the scar tissues [Table 4].

### Histopathological evaluation

**Figure 1:**
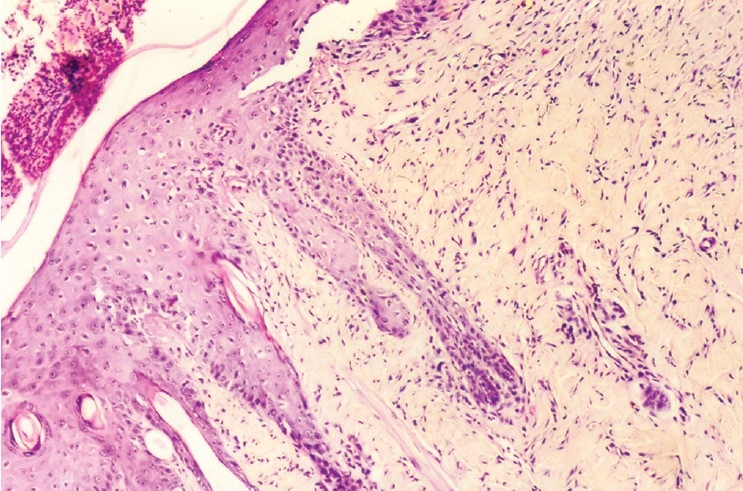
Photomicrograph of 15 day old excision wound of control group

**Figure 2:**
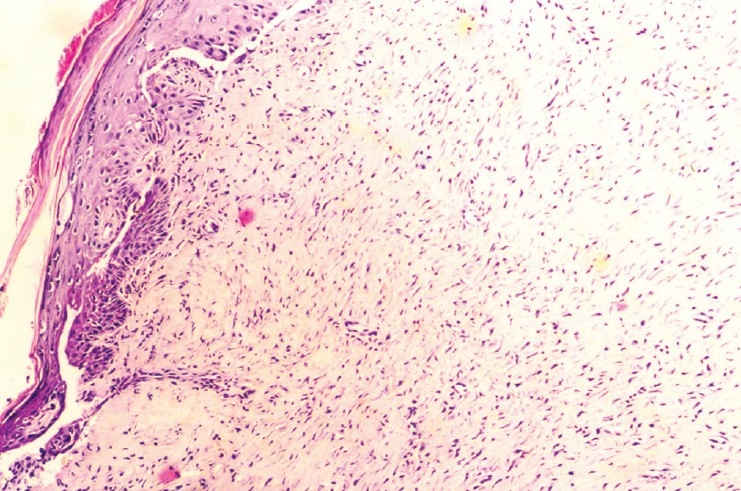
Photomicrograph of 15 day old excision wound of gel base group

**Figure 3:**
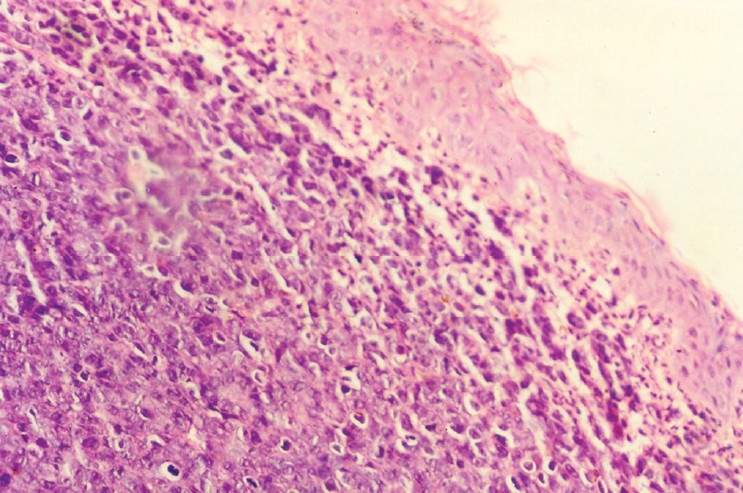
Photomicrograph of 15 day old excision wound of astemizole group

**Figure 4:**
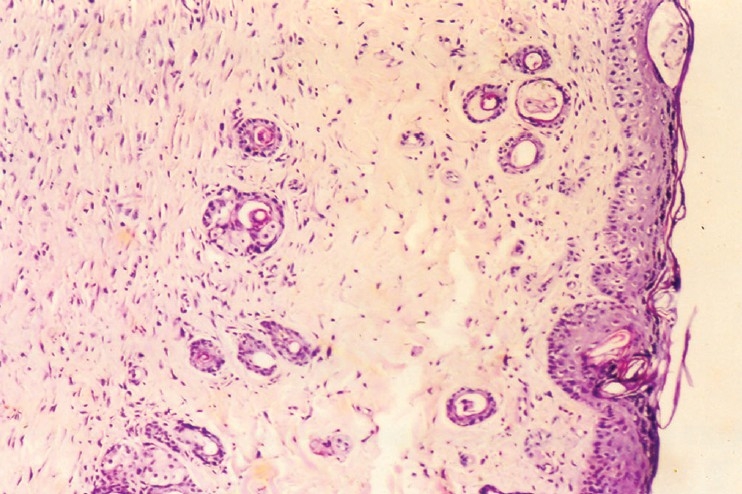
Photomicrograph of 15 day old excision wound of terfenadine group

The wound sections from the animal in control group had a few notable histological observations such as more organized collagen, less number of inflammatory cells and blood vessels – markers of near complete healing. Besides, the growth of epithelial tongues was prominent in these sections [Figure 1]. The features of the sections of the gel base were almost similar to those of control [Figure 2]. On the other hand, the features of the sections of astemizole and terfenadine treated wounds were that of advanced healing, as compared to that of the control or gel base. In these sections, there was complete restoration of epithelial growth, similar to that seen in normal skin. Inflammatory cells appear almost negligible in the dermis [Figures 3 and 4].

The histological features of the sections of astemizole and terfenadine treated wounds confirm the pro-healing actions of these two gels.

## Discussion

Wound is a disruption in the continuity of the living tissues. Restoration of the continuity in a damaged area is brought upon by a house keeping mechanism of the body, viz. the repair process. Tissue repair involves regeneration or replacement or, at times both, leading to the healing of a wound. Itching is one of the problems that the patient faces in the healing of a dermal wound. Histamine causes itching. It needs to be contained or it may hamper the healing process or may cause damage to the scar. Doxepin, an antidepressant having antihistaminic activity upon topical use, has been shown to reduce the itching in burn wounds and scars.[2] However, its influence on healing has not been studied. No treatment either systemic or local (including ointment bases, dressing and suture materials etc.) could be considered inert on the healing process.[1]

In the light of above, the present study was undertaken to see if topical application of antihistaminic drugs could influence healing. Accordingly, gels of astemizole and terfenadine were prepared and tested for their influence on collagenation, wound contraction and epithelization phases of wound healing, upon topical application to incision and excision wounds. The gels of both astemizole and terfenadine promoted healing in both wound models by influencing collagenation, wound contraction and epithelization phases. While the phase of collagenation gives the required strength to the scars of wounds healed by primary and secondary intentions, wound contraction reduces the gap of the open wound to be filled by extracellular matrix (rich in collagen) and finally covered by epithelium.

Two principal components of collagenation phase are collagen synthesis and maturation. Based on the results of the study, it could be assumed that terfenadine and astemizole might have enhanced the strength of the scar by increasing the collagen levels, which could stitch the wound edges together at the repaired site. However, a number of phases of healing, especially coagulation, inflammation, macrophagia, fibroplasia collagenation, wound contraction and epithelization etc. are intimately interlinked. Therefore, a treatment could influence the healing of a wound by intervening in any one or many phases of healing. Thus, based on the present design, it is very difficult to comment on the exact locations and mechanisms of the pro-healing actions of the topical applications. It is quite possible that the drugs could have had a direct influence on the healing, not withstanding their antihistaminic property. For instance, the base of the gel, while it was inert on the collagenation and epithelization phases, promoted contraction during the first ten days. These kinds of differential effects of bases and drug treatments are known to occur.[9]

In conclusion, the study reported a new use for drugs such as terfenadine and astemizole. These drugs produce systemic cardiac toxicity following administration in conventional doses. The gels of these drugs could be free from these effects, as the drug concentration reaching systemic circulation from the topical 1% gel applications would be negligible. These gels may, therefore, have the potential to become useful alternatives to systemic antihistaminics, to reduce healing associated itch and to promote healing of wounds in wound care programs.
